# Relationship between electrocardiogram‐based features and personality traits: Machine learning approach

**DOI:** 10.1111/anec.12919

**Published:** 2021-11-27

**Authors:** Tanja Boljanić, Nadica Miljković, Ljiljana B. Lazarevic, Goran Knezevic, Goran Milašinović

**Affiliations:** ^1^ School of Electrical Engineering University of Belgrade Belgrade Serbia; ^2^ Tecnalia Serbia Ltd. Belgrade Serbia; ^3^ Institute of Psychology and Laboratory for Research of Individual Differences University of Belgrade Belgrade Serbia; ^4^ Department of Psychology and Laboratory for Research of Individual Differences Faculty of Philosophy University of Belgrade Belgrade Serbia; ^5^ Faculty of Medicine University of Belgrade Belgrade Serbia

**Keywords:** disintegration, ECG, HEXACO, machine learning, personality traits, random forest

## Abstract

**Background:**

Based on the known relationship between the human emotion and standard surface electrocardiogram (ECG), we explored the relationship between features extracted from standard ECG recorded during relaxation and seven personality traits (Honesty/humility, Emotionality, eXtraversion, Agreeableness, Conscientiousness, Openness, and Disintegration) by using the machine learning (ML) approach which learns from the ECG‐based features and predicts the appropriate personality trait by adopting an automated software algorithm.

**Methods:**

A total of 71 healthy university students participated in the study. For quantification of 62 ECG‐based parameters (heart rate variability, as well as temporal and amplitude‐based parameters) for each ECG record, we used computation procedures together with publicly available data and code. Among 62 parameters, 34 were segregated into separate features according to their diagnostic relevance in clinical practice. To examine the feature influence on personality trait classification and to perform classification, we used random forest ML algorithm.

**Results:**

Classification accuracy when clinically relevant ECG features were employed was high for Disintegration (81.3%) and Honesty/humility (75.0%) and moderate to high for Openness (73.3%) and Conscientiousness (70%), while it was low for Agreeableness (56.3%), eXtraversion (47.1%), and Emotionality (43.8%). When all calculated features were used, the classification accuracies were the same or lower, except for the eXtraversion (52.9%). Correlation analysis for selected features is presented.

**Conclusions:**

Results indicate that clinically relevant features might be applicable for personality traits prediction, although no remarkable differences were found among selected groups of parameters. Physiological associations of established relationships should be further explored.

## 1 INTRODUCTION

Electrocardiography (ECG) is a non‐invasive clinical technique for monitoring electrical heart activity in cardiovascular diagnostics. Recently, the rich collection of non‐traditional applications of ECG‐based parameters emerged despite partial or incomplete comprehension of their relevance (Chen, 2018).

In this study, we explored the relationship between ECG‐based parameters and personality traits, that is, stable patterns of emotion, motivation, cognition, and behavior (DeYoung, 2015). The most influential, contemporary models of personality postulate the existence of five (McCrae et al., 2005), six (HEXACO, Ashton et al., 2004), or seven broad traits (recently proposed by some authors, such as (Ashton & Lee, 2020; Knezevic et al., 2017)) subsuming many narrower ones in the lower level of hierarchy. These traits are found to be universal in humans (McCrae et al., 2005) and subhuman species (Gosling & John, 1999), longitudinally stable, with about 40% of their variability heritable (Vukasović & Bratko, 2015). Available evidence indicates that personality traits have profound relationships with peripheral physiology. A modular influence of brain structures implicated in personality traits, such as orbitofrontal and insular cortex, amygdala, hippocampal formation, and hypothalamus (Deckersbach et al., 2006; Depue & Collins, 1999; Koelsch et al., 2007; Panksepp, 1998), seems to be responsible for these relationships. In addition, data show connections between personality traits and peripheral organs and tissues through the autonomic, endocrine, and immune systems (Cloninger, 2000; Depue & Collins, 1999; Irwin, 2008). Therefore, due to the well‐known and established influence of the autonomic nervous system on ECG, finding the connection between ECG signal and personality traits seems promising.

Available evidence showed that heart rate decreases and heart rate variability (HRV) increases with Extraversion (Brouwer et al., 2013), Neuroticism correlates with QT interval (Minoretti et al., 2006), and Agreeableness correlates with P, QRS, and T amplitude (Koelsch et al., 2012). Typically, the relationship between personality traits and physiological measures is investigated descriptively, that is, using correlations (Koelsch et al., 2007) or by trying to predict cardiac output with scores on personality questionnaires.

We used the supervised machine learning (ML) approach to examine this relationship. ML is a computer algorithm that automatically assigns traits to the input set of ECG‐based features by going through the training and testing phase. The training phase is used for constructing an optimal model that learns from the available ECG features and corresponding traits, while the testing phase is used to evaluate ML performance. Here, we adopted random forest (RF) ML algorithm for trait classification and feature selection as it achieved high prediction accuracy in similar ECG‐based investigations (Dissanayake et al., 2019; Melillo et al., 2015) and it is suitable for processing a large number of variables with complex interactions (Breiman, 2001; Strobl et al., 2009).

Random forest ML was applied on ECG‐based features with proven clinical efficacy in diagnostics, that is, clinically relevant features (Electrophysiology, 1996; Wagner et al., 2008) and on other parameters due to their attractive and practical characteristic as they are calculated from the local ECG extremes being more robust to noise than standard clinically relevant parameters (Arteaga‐Falconi et al., 2016; Cabra et al., 2018) and have proven efficacy in previous studies (Cabra et al., 2018; Israel et al., 2005; Sansone et al., 2013; Shen et al., 2010).

### 1.1 Aim of the study

We test a novel approach for extracting ECG‐based features related to personality traits with RF ML algorithm applied on 62 ECG‐based parameters and investigate perceptible changes within intervals of parameters in healthy individuals, to detect the possible relationships between ECG and individual differences in personality traits. An exploratory analysis of ECG‐based feature selection is presented.

## 2 METHODS AND MATERIALS

Electrocardiogram data analyzed in this study were recorded for another project aiming to investigate emotions and affects by the means of physiological measurements (Bjegojević et al., 2020). We used baseline recording of 120‐s long ECG segment recorded in sitting position before the emotion induction to avoid subjects’ emotion influence.

### 2.1 Study sample

The sample consisted of 71 university students, average age 20.38 years (*SD* = 2.96), 78.8% female. Exclusion criteria were previous cardio‐vascular disorders. The study has been approved by the Institutional Review Board of the Department of Psychology, University of Belgrade No. 2018‐19. Respondents signed informed consents in accordance with the Declaration of Helsinki.

### 2.2 Assessment of personality traits

The HEXACO Personality Inventory‐Revised HEXACO PI‐R (Lee & Ashton, 2018) contains 100 items with a 5‐point Likert‐type scale ranging from 1 (strongly disagree) to 5 (strongly agree). It assesses six personality domains: Honesty/humility, Emotionality, eXtraversion, Agreeableness, Conscientiousness, and Openness. We used the Serbian form of HEXACO PI‐R (Međedović et al., 2019). Domain scores were calculated as the average scores on all items mapping specific domains (ranging from 1 to 5). The Disintegration trait was measured via a DELTA questionnaire containing 110 items with the same 5‐point Likert‐type scale. The score is calculated as the average of scale items (also ranging from 1 to 5).

### 2.3 Recording procedure

Upon arrival, all respondents were introduced to the study and fitted the BIOPAC sensors (Biopac Systems Inc.) (Bjegojević et al., 2020). Subjects were seated and instructed to relax with eyes open and to avoid movements as much as possible to reduce the artifacts. ECG signals were visually inspected for quality on site. All subjects were blinded for the ECG signal and related parameters. Personality measures were collected separately, before physiological measurements.

Electrocardiogram signals were recorded from standard bipolar Lead I using the BIOPAC MP150 unit with AcqKnowledge software and ECG 100C module with surface H135SG Ag/AgCl electrodes (Kendall/Covidien). Before electrode placement, the skin was cleaned with Nuprep gel (Weaver & Co.) to reduce skin–electrode impedance. The sampling frequency was set at 2000 Hz.

### 2.4 ECG preprocessing and feature extraction

The complete procedure of ECG preprocessing and feature extraction is described in Boljanić et al. (2021). Computed ECG peak locations and corresponding absolute peak amplitudes were employed for extracting three groups of clinically relevant and clinically non‐relevant features based on the HRV, temporal parameters, and relative amplitude.

We used three domains to calculate HRV‐based features: time, frequency, and geometry. The overview of HRV‐based features is displayed in Table 1 together with the relevant references related to its application and calculation. All HRV‐based features were classified as clinically relevant features, except for the HRV index, as it has been defined and consequently used for 24‐h ambulatory ECG monitoring and not for short‐term recordings of 2‐min duration as applied here (Cripps et al., 1991; Kouidi et al., 2002). Therefore, we applied RF ML on all features with and without the HRV index.

**TABLE 1:** Heart rate variability (HRV)‐based features for three feature domains (time, frequency, and geometry) with corresponding units and related references

Feature	Feature domain	Unit	References	Description
HR mean	Time	bpm	Abadi et al. (2015), Kim and Andre (2008), Tulppo et al. (1996)	Average heart rate
RR mean	Time	s	Abadi et al. (2015), Dissanayake et al. (2019), Electrophysiology (1996), Kim and Andre (2008)	Average of all RR intervals
rmssd	Time	s	Abadi et al. (2015), Abbasi (2004), Dissanayake et al. (2019), Electrophysiology (1996), Kim and Andre (2008)	Root mean square of all RR intervals
sdnn	Time	s		Standard deviation of all RR intervals
m_nn	Time	s	Abadi et al. (2015), Dissanayake et al. (2019), Kim and Andre (2008), Koelsch et al. (2012)	Maximal RR interval
nn50	Time	count	Abadi et al. (2015), Abbasi (2004), Dissanayake et al. (2019), Electrophysiology (1996), Kim and Andre (2008)	Number of pairs of adjacent RR intervals differing by more than 50 ms in the entire recording
pnn50	Time	%		nn50 count divided by the total number of all RR intervals
sdsd	Time	s	Abadi et al. (2015), Abbasi (2004), Electrophysiology (1996), Kim and Andre (2008), Tulppo et al. (1996)	Standard deviation of differences between adjacent RR intervals
HRV index	Time	n.u.	Abbasi (2004), Cripps et al. (1991), Electrophysiology (1996), Kouidi et al., 2002)	HRV triangular index ‐ integral of the density distribution (the number of all RR intervals) divided by the maximum of the density distribution at a discrete scale of 1/*fs* bins, where *fs* is a sampling frequency
LF	Frequency	s^2^	Abadi et al. (2015), Abbasi (2004), Dissanayake et al. (2019), Electrophysiology (1996), Kim and Andre (2008), Koelsch et al. (2012), Tulppo et al. (1996)	Spectral power of low frequency (0.04–0.15 Hz)
HF	Frequency	s^2^		Spectral power of high frequency (0.15–0.40 Hz)
LFHF	Frequency	n.u.	Abbasi (2004), Dissanayake et al. (2019), Electrophysiology (1996), Kim and Andre (2008), Koelsch et al. (2012), Tulppo et al. (1996)	LF to HF ratio
LFnu	Frequency	n.u.	Abbasi (2004), Dissanayake et al. (2019), Electrophysiology (1996)	LF in normalized units in relation to the total power without very low frequencies
HFnu	Frequency	n.u.		HF in normalized units
Total power	Frequency	s^2^		Total PSD power
SD1	Geometry	s	Dissanayake et al. (2019), Kim and Andre (2008), Koelsch et al. (2012)	Length of the transverse line of the Poincaré plot in the perpendicular direction. Poincaré plot presents a scatter plot of the current RR interval in relation to the prior RR interval.
SD2	Geometry	s		Length of the longitudinal line of the Poincaré plot in the perpendicular direction

Abbreviations: bpm, beats per minute; n.u., no unit.

The overview of extracted temporal features is displayed in Table 2.

**TABLE 2:** Temporal features—clinically relevant and clinically not relevant parameters with normal values and ranges where applicable

Distance	Description	Features	References	Normal range (s)
PR	Measured from the fiducial point P to the R peak	PR_min, PR_max, PR_mean, PR_median, PR_sd	Cabra et al. (2018), Dissanayake et al. (2019)	Na
ST	Measured from the fiducial point S to the fiducial point T	ST_min, ST_max, ST_mean, ST_median, ST_sd		Na
QRS	Measured from the fiducial point Q to the fiducial point S	QRS_min, QRS_max, QRS_mean, QRS_median, QRS_sd		Na
PR interval^a^	Measured from the beginning of the P wave to the beginning of the QRS complex	PRinterval_mean, PRinterval_sd	Wagner et al. (2008)	0.12–0.20
PR segment^a^	Measured from the end of the P wave to the beginning of the QRS complex	PRsegment_mean, PRsegment_sd		0.05–0.12
ST interval^a^	Measured from the end of the QRS complex to the end of the T wave	STinterval_mean, STinterval_sd		0.42
ST segment^a^	Measured from the end of the QRS complex to the beginning of the T wave	STsegment_mean, STsegment_sd		0.005–0.150
QRS complex^a^	Measured from the beginning of the QRS complex to the end of the QRS complex	QRScomplex_mean, QRScomplex_sd		0.08–0.12
P wave^a^	Measured from the beginning of the P wave to the end of the P wave	Pwave_mean, Pwave_sd		≤0.12
T wave^a^	Measured from the beginning of the T wave to the end of the T wave	Twave_mean, Twave_sd		0.10–0.25
QTc interval^a^	Measured from the beginning of the QRS complex to the end of the T wave and compensated according to Bazzet's formula	QTnorm_mean, QTnorm_sd		Men: <0.45 Women: <0.46 0.35–0.43 (QT)

Abbreviations: na, not available; QTc, corrected QT interval; Suffixes _min, _max, _mean, _median and _sd stand for minimal value, maximal value, mean, median, and standard deviation, respectively.

a Clinically relevant parameters. Bazzet’s formula:$\text{QTc}\;=\;\text{QT}/\sqrt{\text{RR}}$.

The overview of extracted amplitude‐based features is displayed in Table 3. The Ek parameter has been suggested as a cardiac signature of emotionality and personality in previous studies (Koelsch et al., 2007, 2012). It presents a weighted linear relation of ECG amplitudes unrelated to the person’s BMI with a direct correlation with Emotionality. Thus, higher Ek indices correspond to higher Emotionality measured by the Revised Toronto Alexithymia Scale (Taylor et al., 1992) and vice versa. Originally, Ek indices are determined from the 12‐lead resting ECG (Koelsch et al., 2007, 2012). By carefully studying the proposed Ek and its practical significance (BMI and electrode positioning compensations), we concluded that Ek can be calculated for one‐channel ECG.

**TABLE 3:** Amplitude‐based ECG parameters

Distance	Feature (n.u.)	References	Description
PRa	PRa_mean, PRa_sd	Cabra et al. (2018), MNUA	Relative amplitude differences between P and R
RQa	RQa_mean, RQa_sd	Arteaga‐Falconi et al. (2016), Cabra et al. (2018)	Relative amplitude differences between R and Q
RSa	RSa_mean, RSa_sd		Relative amplitude differences between R and S
RTa	RTa_mean, RTa_sd	Cabra et al. (2018), MNUA	Relative amplitude differences between R and T
Sta	STa_mean, STa_sd		Relative amplitude differences between S and T
QSa	QSa_mean, QSa_sd		Relative amplitude differences between Q and S
Ek	Ek_mean, EK_sd	Koelsch et al. (2007, 2012)	Calculating formula is available in Boljanić et al. (2021)

Abbreviations: MNUA, Mentioned in literature not used for analysis; n.u. no unit.

**FIGURE 1:**
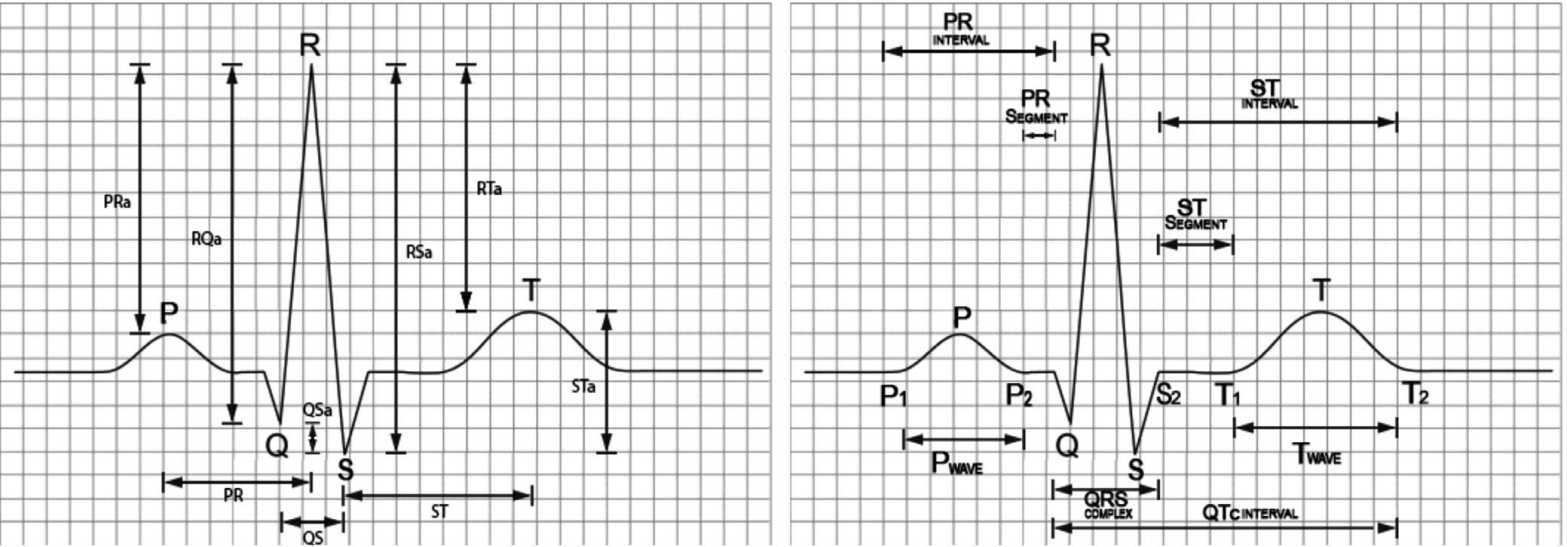
Normal heartbeat ECG signal marked with temporal and amplitude‐based features: clinically not relevant (left‐hand panel) and clinically relevant (right‐hand panel) parameters

The ECG signal with marked time distances and amplitude differences is shown in Figure 1.

### 2.5 Analytic strategy

**FIGURE 2:**
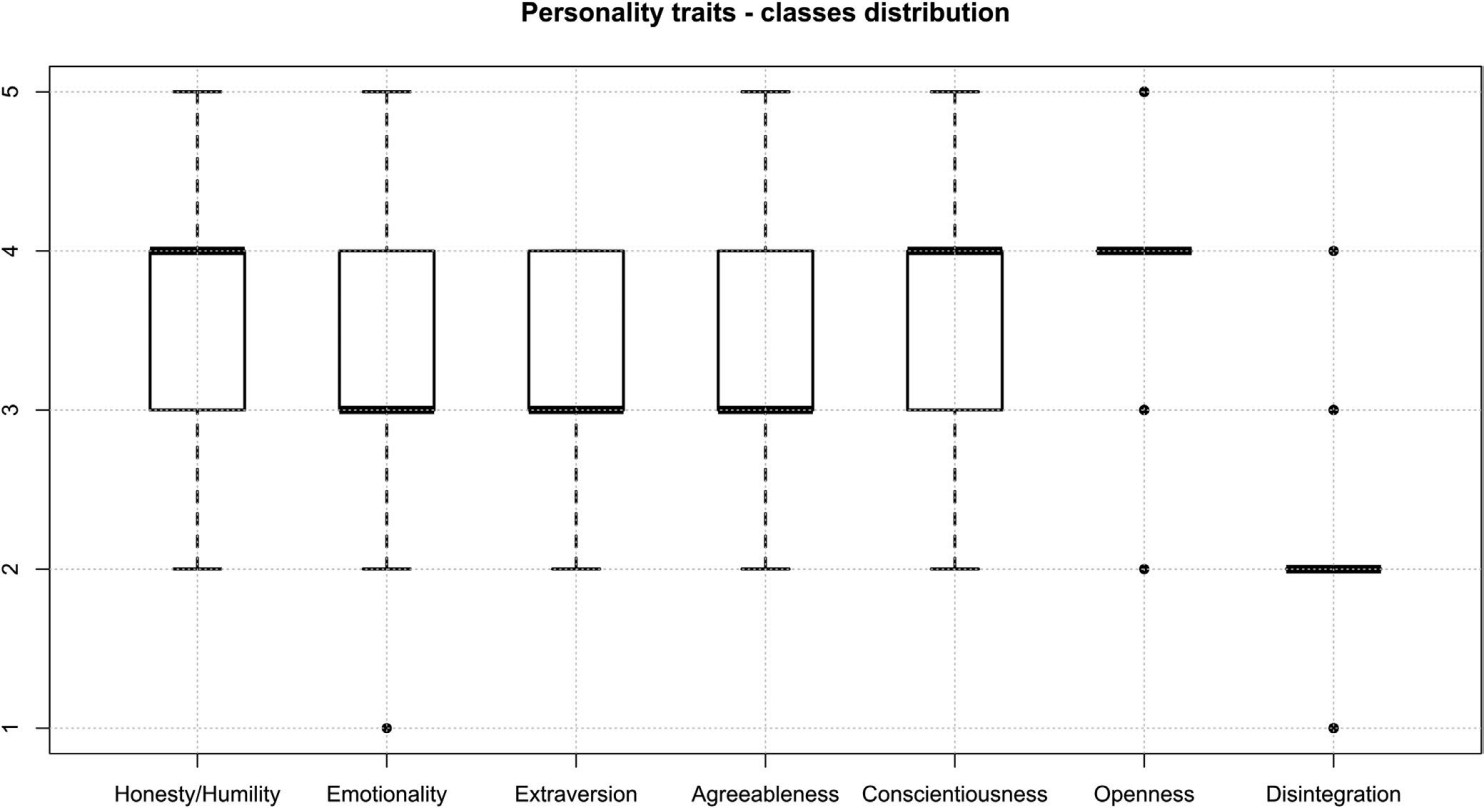
Distribution of five categories of personality traits for 70 subjects presented with box plots

We applied RF ML separately for each personality trait. As psychological test results ranged from 1 to 5, to perform classification and test our hypothesis on a more distinctive personality scores grouping, we used the following reasoning for splitting data: 1 for 1.00–1.50, 2 for 1.51–2.50, 3 for 2.51–3.50, 4 for 3.51–4.50, and 5 for 4.51–5.00. The distribution of classes is presented in Figure 2.

Random forest is an ensemble ML algorithm, consisting of basic models called decision trees where the predictions of all individual trees are combined. Each tree returns a predicted class for the same classification problem and the class that most trees vote for is returned as the prediction of the ensemble and as the final outcome of the algorithm. RF also enables the calculation of feature importance by counting the number of times each variable is selected by all individual trees in the ensemble termed feature importance. Unlike other nonlinear classifiers, RF ML is robust to over‐fitting (working perfectly well on a small dataset and poorly on a more general dataset) and yields good classification results even without extensive tuning of the algorithm parameters (Breiman, 2001; IJzerman et al., 2016; Shen et al., 2007; Zhou et al., 2019). RF ML was also used to estimate variable importance.

Parameters were split into three groups and RF was applied on all parameters with (62 overall) and without HRV index (61), and on clinically relevant parameters (34). By clinically relevant parameters, we observed HRV‐based features except for the triangular index (16), temporal features (8 × 2), and Ek (2). Each dataset was divided into a training and a testing set (75% and 25% of data, respectively (Attia et al., 2019)). We used R function *createDataPartition* that randomly splits the data taking into the class distribution balance. We further applied 10‐fold cross‐validation on the training set using *trainControl* function that provided an overall accuracy estimate (Ross et al., 2009).

For RF ML application, we tuned decision trees used in the forest (*ntree*) and random variables used in each decision tree (*mtry*) by application of tuning *Caret* procedure to minimize parameters effect on the final accuracy (Brownlee, 2016). We reported mean classification accuracies and confident intervals.

For personality traits with accuracies ≥75%, the first 10 feature importances were plotted for three sets of parameters. We used the *varImp* function from the *Caret* package for ranking features by importance. Furthermore, to assess the degree of association between the test scores (both original and mapped into categories) and the top 10 features as in Melillo et al. (2015), we used the Spearman correlation coefficient and calculated the statistically significant correlations as suggested before (Koelsch et al., 2007; Minoretti et al., 2006). *p* Values were set to .05, .01, and .001.

## 3 RESULTS

Descriptive statistics for all personality measures are shown in Table 4.

**TABLE 4:** Descriptive statistics (*N* = 71)

Personality traits	*M*	*SD*	Range	Skew	Kurt
Honesty/Humility	3.57	0.66	1.69–4.88	−0.54	2.98
Emotionality	3.47	0.67	1.44–4.88	−0.22	3.24
Extraversion	3.34	0.71	1.56–4.50	−0.37	2.79
Agreeableness	3.13	0.71	1.63–4.75	−0.22	2.72
Conscientiousness	3.67	0.67	1.88–4.94	−0.42	2.67
Openness	3.85	0.59	1.81–4.88	−0.97	4.14
Disintegration	2.07	0.50	1.10–3.81	0.97	4.87

Abbreviations: Kurt, kurtosis; *M,* mean; *SD*, standard deviation; Skew‐skewness.

In Table 5, mean classification accuracies when 10‐fold cross‐validation of RF ML algorithm was performed with 95% confident intervals for all seven personality traits when all features and only clinically relevant features were used are presented. Classification accuracies for the special case (without HRV index) are also presented (Table 5).

**TABLE 5:** Mean classification accuracies for personality traits using all features and only clinically relevant

Trait	All features (62)		All features without HRV index (61)		Clinically relevant features (34)	
	Mean accuracy [%]	95% Confident interval	Mean accuracy [%]	95% Confident interval	Mean accuracy [%]	95% Confident interval
Honesty/Humility	75.0	47.6–92.7	75.0	47.6–92.7	75.0	47.6–92.7
Emotionality	31.3	11.0–58.7	37.5	15.2–64.6	43.8	19.8–70.1
Extraversion	35.3	14.2–61.7	52.9	27.8–77.0	47.1	23.0–72.2
Agreeableness	56.3	29.9–80.3	50.0	24.7−75.4	56.3	29.9–80.3
Conscientiousness	64.7	38.3–85.8	58.8	32.9–81.6	70.6	44.0–89.7
Openness	73.3	44.9–92.2	73.3	44.9–92.2	73.3	44.9–92.2
Disintegration	81.3	54.4–96.0	81.3	54.4–96.0	81.3	54.4–96.0

Note

95% confidence intervals are presented for single classification accuracy. With mean accuracies, 10‐fold cross‐validation results of random forest classifiers are presented.

**FIGURE 3:**
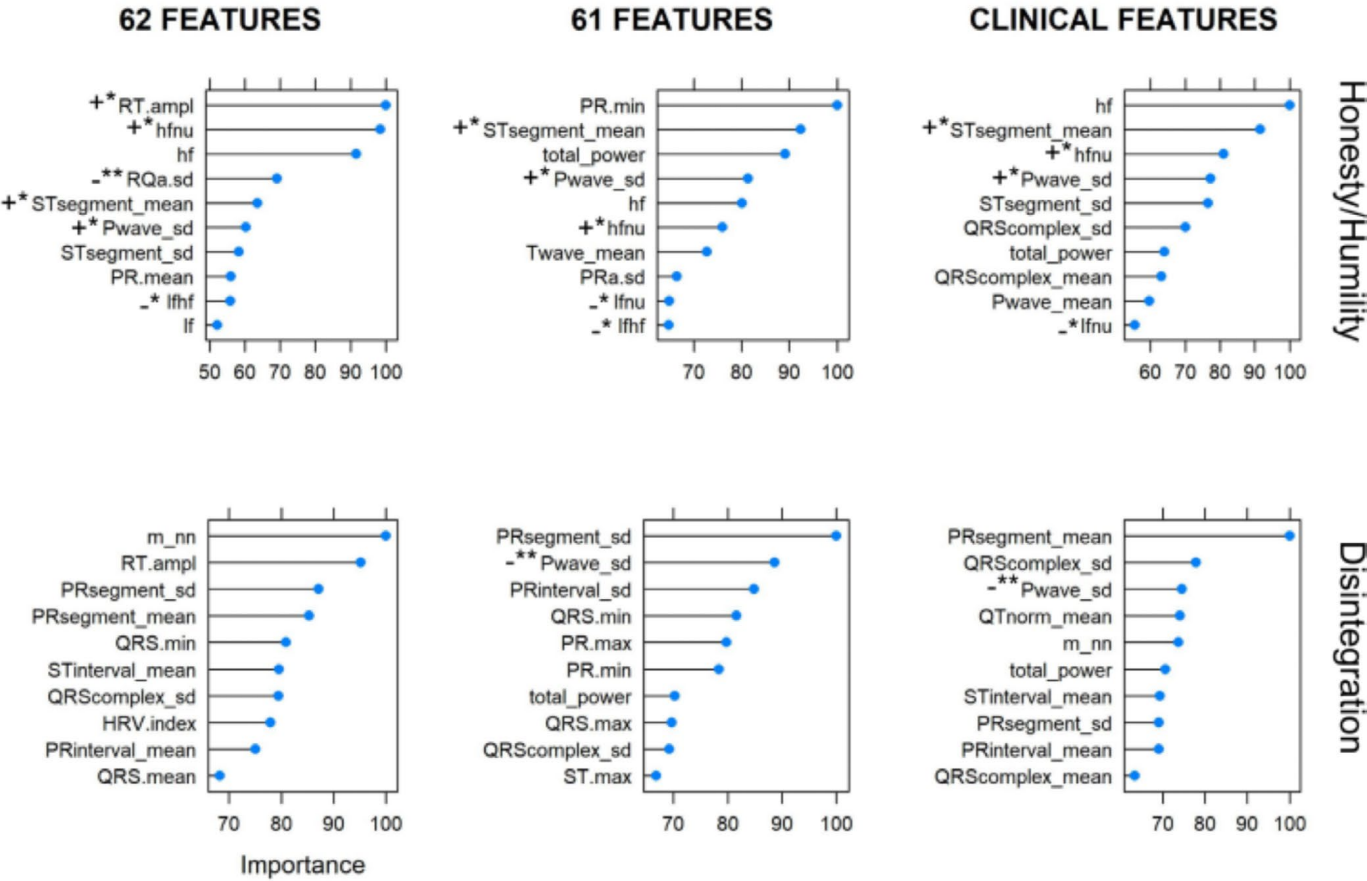
Top 10 feature importances for Honesty/humility and Disintegration: all 62 features (left‐hand panel), all features without HRV index (middle panel), and clinically relevant features (right‐hand panel). NOTE: Feature importances are normalized to 100. Statistically significant Spearman correlations are presented with *p < .05, and with **p < .01. Sign – presents negative correlation and sign +positive correlation. For feature abbreviations, please see Section 2

The top 10 feature importances are presented for Disintegration and Honesty/humility in Figure 3. Statistically significant Spearman correlations between scores of personality traits and top 10 features are reported in Figure 3 together with the correlation sign. Only statistically significant correlations with *p* < .05 and *p* < .01 were found (Figure 3). For Disintegration, significant (*p* < .05) negative correlations were found between categories and QTnorm_mean (−0.203) and HRV.index (−0.245), while significant positive correlations were found for RT.ampl (0.335) and m_nn (0.279) being partly in line with score classification in Figure 3. For Honesty/humility, statistically significant correlations (*p* < .05) were found between trait categories and lfnu (−0.270), lfhf (−0.273), RQa.sd (−0.224), and hfnu (0.210).

## 4 DISCUSSION

In presented study, RF ML approach success varied across personality traits: from 31.3% (being less than the probability of coin flips) for Emotionality to 81.3% for Disintegration (Table 5). The highest classification accuracy was obtained for Disintegration (mean accuracy of 81.3%) and for Honesty/humility (75.0%) for all feature sets (Table 5). This “robust” result to the feature set might be the consequence of the distribution of subjects across categories for traits in Figure 2 (we assessed personality traits only in university students, known to have higher Openness and lower Disintegration).

We used a considerably large list of features providing a more general approach by selecting the most influencing features. Our feature list is exhaustive, and there are many correlated features such as hf and hfnu, so the feature importance list based solely on RF should be taken with precaution. Previous studies have shown that multicolinearity does not affect the classification accuracy^1^, but does influence feature importances (Strobl et al., 2008; Toloşi & Lengauer, 2011). This is in line with our results as the feature importance instability is visible in Figure 3 for 61 and 62 parameters. We used both Spearman correlation coefficients and importance plots to discuss selected features appropriately.

Recently, QT variability index (QTVI) was previously compared with Anger and Hostility traits in patients with implantable cardioverter defibrillator patients had significantly higher QTVI than controls (Krantz et al., 2021). We found a statistically significant negative correlation between the Disintegration category and QTnorm_mean which was not expected as QT interval duration which reflects the time for ventricular recovery increases with Neuroticism (Minoretti et al., 2006). This disagreement might be a consequence of categories distribution (Figure 2), which could have caused a spurious correlation. More likely, these discrepancies might be the consequence of the different methods to assess Emotionality: They used NEO^2^ personality inventory (NEO PI‐R) (Costa, 1992) to assess Neuroticism, which is conceptually close to Emotionality used here (Fearfulness and Anxiety), but there are important differences: Neuroticism in NEO PI‐R has contents related to low Agreeableness (Angry Hostility, and Impulsiveness) and Depression, while Emotionality contains aspects characterizing agreeable persons (Dependability and Sentimentality) without Depression (Ashton et al., 2004). Alternatively, the fact that Neuroticism correlates to some extent with Disintegration (Knežević et al., 2016) might also explain this discrepancy. Though prolongation of QT interval is associated with a variety of acute and chronic cardio‐vascular conditions (Campbell et al., 1985), its relationship with personality traits should be further explored.

Another interesting parameter is the HRV index that appeared among the top 10 features for Disintegration (Figure 3), but it was not statistically correlated with this trait. It could be discussed whether the HRV index was calculated properly or it influenced the importance plot as a garbage feature. Interval of 120 s was sufficient for all calculated parameters except for the HRV index being commonly calculated for Holter recordings (Cripps et al., 1991; Kouidi et al., 2002). More in‐depth analysis in our study revealed that the HRV index was statistically related (positive correlation of .281) to the Extraversion, but it showed a poor classification accuracy of 35.3%. Decreased HRV index has been associated with higher risks of cardiovascular death in patients with atrial fibrilation (Hämmerle et al., 2020) with a possible meaning that less extroverted persons would be more prone to cardiovascular diseases. Decreased HRV index can be, therefore, useful for risk stratification as a measure of sympathovagal balance. Relationship of Introversion with HRV index and its risk in healthy respondents is yet to be established.

Further analysis of obtained results showed that Disintegration (Figure 3) was characterized by variation in P wave—it was negatively correlated with the Pwave_sd (−.107). On the contrary, we found that Honesty/humility was positively correlated with Pwave_sd (.196), which is not surprising given the negative correlation between Honesty and Disintegration. P wave reflects atrial conduction delay, and multivariate logistic regression analysis revealed that it is significantly longer in patients with atrial fibrillation (Steinberg et al., 1993). This is probably a consequence of depressed conduction that resulted in prolonged atrial activation and loner P wave. P wave variation was associated with atrial fibrillation in patients (Censi et al., 2016). Higher variability in the P wave indicates changes in atrial conduction, and we can only speculate whether it presents a risk factor for atrial fibrillation in healthy subjects with higher Honesty/humility and lower Disintegration scores.

For Honesty/humility, we identified in the current study the following important clinical features with positive correlation STsegment_mean (.239), hfnu (.231), and Pwave_sd (.196), while negatively correlated clinical features were lfnu (−.280) and lfhf (−.278). The prolonged ST segment is related to the increased Honesty/humility score. ST segment presents interval between ventricular depolarization and repolarization. Prolonged ST segment in the absence of Q wave in a case study was related to the heart tumor (Hartman, 1982). However, there is no stronger evidence on psychophysiological bases of ST duration. As lfnu, hfnu, and lfhf are interrelated, the positive correlation with hfnu and negative with lfnu and lfhf were expected. Lfhf ratio reflects the autonomic balance of the sympathetic and parasympathetic parts of the autonomic nervous system, and it has been shown that maturity (being self‐directed, cooperative, and self‐transcendent) was negatively associated with the lfhf (Koelsch et al., 2012; Zohar et al., 2013). As Honesty/humility assumes more mature behavior, our finding on the negative correlation between Honesty/humility and lfhf is in line with the previous ones. Higher hf was also found in individuals that were more sensitive to positive states of others indicating more successful maintaining of social relationship with pronounced parasympathetic activity (Lischke et al., 2017).

Overall, though physiological basis of adoption of clinically relevant parameters exist, the exact and the most influential parameters in relation to specific personality trait are yet to be discovered as the current base of knowledge is vastly related to clinical conditions. We believe that this study provides a perspective in ECG‐based features potential for studying personality traits in relation to ECG parameters changes within healthy ranges, as well as for further investigation personality traits in individuals with cardio‐vascular diseases. Once the relationships are clearly determined, we may be able to answer whether individual traits present risk factor for cardio‐vascular condition or vice versa, or the relationship is of a different origin and complexity.

For nonclinical features, we identified positive correlations of Honesty/humility with RT.ampl (.135) and negative with RQa.sd of −.324 (Figure 3). RT.ampl and RQa.sd have been previously used and proposed for person identification. No known physiological basis for their explanation exists, although we observed that a higher R peak concerning the T peak and lower variability of R and Q peaks yields to increased Honesty/humility. Distances between local extrema on ECG signal are termed amplitude and temporal distances (Arteaga‐Falconi et al., 2016; Cabra et al., 2018; Israel et al., 2005), and though there is no clear clinical rationale for the application of these parameters, we computed them due to the demonstrated results (Shen & Tompkins, 2005). Our results (Table 5) suggest that Conscientiousness classification benefits the most from the sole application of clinically based parameters with an increase from 9.1% to 20.1%. The unreserved advantage of clinical features is in their proven relation to physiological processes, but the potential of clinically not relevant features should not be forsaken.

We identified the following limitations of the study:

1. We used RF ML due to its proven efficiency for emotion recognition and prediction of cardiovascular events when classifying ECG‐based features (Dissanayake et al., 2019; Melillo et al., 2015). A careful selection of the most appropriate algorithm should be performed.
2. Additional data from the general population and especially from an independent cohort are needed for further confirmation of presented associations between ECG‐based parameters and personality traits, although our results present a firm base for future research as changes detected in the student sample should be more prominent in the general population. For future comparisons and meta‐analysis, the dataset used in this study is publicly available (Boljanić et al., 2021).
3. Results presented by RF ML cannot provide exact relation direction (positive and/or negative) and additional approaches are needed to distinguish the exact relation of cardiac parameters and personality.
4. In this stage of understanding of the relationships between disposition‐based behavioral regularities (personality) and cardiac parameters, we consider a prediction‐focused approach based on ML of exceptional importance. Premature and incorrect explanatory conclusions appearing to be simple and elegant were shown to have detrimental effects on the development of a scientific field. It was shown that good predictive models based on machine learning can improve our understanding of such relationships (Yarkoni & Westfall, 2017)—prior to focusing on the precise underlying neural mechanisms.

## 5 CONCLUSIONS

The main contribution is an enhanced body of knowledge regarding the relationships between ECG‐based features and personality traits (HEXACO model complemented with Disintegration trait) based on a novel analytical strategy—machine learning.

Random forest ML and Spearman’s correlations allowed us to formulate associations out of a large number of ECG‐based features indicating the following statements that should be re‐confirmed:

1. higher Honesty/humility is directly related to the lower lfhf ratio suggesting that more mature behavior and fairness in dealing with others is related to more pronounced vagal tone,
2. less Extraverted persons could be more prone to cardiovascular diseases as revealed by the HRV triangular index, and
3. Disintegration (proneness to psychotic‐like experiences/behaviors) was found to be related to QT interval duration and P wave variance, as well as HRV.

Replication of presented findings especially with the focus on Disintegration and Honesty/humility in an independent cohort would be a highly welcomed first step toward the development of more explanation‐oriented (neural) theories and studies. Our results include open data as well as open and free software for further in‐depth exploratory investigation, replication, and future meta‐analysis (Boljanić et al., 2021).

## CONFLICT OF INTEREST

None.

## AUTHOR CONTRIBUTIONS

Tanja Boljanić: Conceptualization, Investigation, Software, Visualization, Writing–original draft preparation, Nadica Miljković: Conceptualization, Methodology, Supervision, Writing—review and editing, Ljiljana B. Lazarević: Conceptualization, Methodology, Supervision, Writing—review and editing, Goran Knežević: Data curation, Resources, Methodology, Writing—review and editing, Goran Milašinović: Conceptualization, Methodology, Visualization, Writing—review and editing.

## ETHICS STATEMENT

The study was approved by the Institutional Review Board of the Department of Psychology, Faculty of Philosophy at the University of Belgrade (approval number: 2018‐19).

## Supporting information

Supplementary Material

## Data Availability

The raw data that support the study findings were recorded at the Faculty of Philosophy, University of Belgrade, and will be openly available with R programming code for feature extraction in Zenodo public repository under CC Attribution 4.0 International license.

## ENDNOTES

^1^
We reconfirmed this statement as suggested by reviewer. We applied principal component analysis (PCA) prior to RF ML and showed that classification accuracies were higher only in two cases (~6% higher with relatively low resulting classification accuracies of 56.3% and 62%) for all traits and all three datasets when only 10 principal components were used as RF ML input.

^2^
NEO inventory was named after acronym of the three‐factor personality model including Neuroticism, eXtraversion, and Openness personality traits, but now it covers two additional factors Agreeableness and Conscientiousness and is used to present five‐factor model.
