# Supplementary material for: Relationship between electrocardiogram‐based features and personality traits: Machine learning approach
Source: Ann Noninvasive Electrocardiol. 2021 Nov 27;27(1):e12919. doi: 10.1111/anec.12919 (PMC8739611; doi:10.1111/anec.12919)
Supplement: Supplementary file 1 — Supplementary Material [file ANEC-27-e12919-s001.docx]

**NOTE**: This Method for ECG preprocessing and feature extraction is prepared for the paper T. Boljanić, N. Miljković L. B. Lazarević, G. Knežević, G. Milašinović, “Relationship between electrocardiogram-based features and personality traits: Machine learning approach”, submitted for the Annals of Noninvasive Electrocardiology in 2021. Therefore, this material refers to and contains tables and text prepared for the aforementioned paper and will be shared in OA together with data and code.

ECG preprocessing & feature extraction

All processing steps were performed in R programming language version 3.6.2 (Team, 2018) using RStudio IDE (Rstudio, Inc., Boston, USA) with the up-to-date R packages from the Comprehensive R Archive Network (CRAN). We used the following CRAN packages for intermediate and final results presented in this manuscript: dplyr (Wickham et al., 2015), signal (Developers, 2013), pracma (Borchers, 2019), ggplot2 (Hadley, 2016), Hmisc (Harrell, 2017), corrplot (Wei et al., 2017), zoo (Zeileis&Grothendieck, 2005), e1071 (Meyer et al., 2019), caret (Kuhn et al., 2020), randomForest (Paluszynska et al., 2020), PerformanceAnalytics (Peterson et al., 2018), randomForestExplainer (Paluszynska et al., 2020), moments (Komsta&Novomestky, 2015), tibble (Müller & Wickham, 2019), gtable (Wickham & Pedersen, 2019), and gridExtra (Augie et al., 2017).

The ECG signals were filtered with the 3^rd^ order Butterworth low-pass filter with a cut-off frequency of 100 Hz to remove noise dominantly originating from electrical muscle activity. Then, a high-pass filter with a cut-off frequency of 1 Hz was applied to eliminate baseline wander with abrupt drift (Antić et al., 2020; Satija et al., 2018).

After filtering, ECG signal amplitude was normalized according to the recommendation provided in (T. W. Shen & Tompkins, 2005) that enabled comparison of different units and compensation for Body Mass Index (BMI): from each sample in 120 s long interval we subtracted the global minimum (${min}_{global}$) and then divided sample amplitude with the global range as presented in Eq. 1.

| ${sample}_{normalized}= \frac{sample-{min}_{global}}{{max}_{global}-{min}_{global}}$ | Equation (1) |
| --- | --- |

Then, ECG delineation, i.e., detection of characteristic ECG waves was performed. A simple method to find local maxima was used to find the R peak by application of *findpeaks* function from the pracma library in R with two parameters: minimal peak height with a threshold set to 60% of the maximal value that was determined by error and trial. This threshold provided detection of all R peaks with no missed or false detections which were confirmed by visual inspection. The minimal peak distance in *findpeaks* function was set at 300 ms as we assumed that heart rhythm would not exceed 200 bpm (beats per minute) during relaxation. We performed an ensemble average to eliminate noise and to provide an adequate ECG delineation as proposed for ECG signal processing (*ECG Analysis Using the Offline Averaging Mode*, 2006; Kligfield et al., 2006; Koelsch et al., 2007, 2012; Minoretti et al., 2006). Ensemble averaging was applied on 10 ECG complexes that were aligned in relation to the R peak as it is less sensitive to noise (Antić et al., 2020; Arteaga-Falconi et al., 2016; Cabra et al., 2018).

TABLE 1 Parameters for detection of the ECG fiducial points in respect to R peak and consecutive peaks

| Fiducial point | Q | S | P |  | T | P1 | P2 | T1 | T2 | S2 |
| --- | --- | --- | --- | --- | --- | --- | --- | --- | --- | --- |
| Detection | Min before R peak | Min after R peak | Max before Q peak |  | Max after S peak | Min before P peak | Min after P peak | Min before T peak | Min after T peak | Max after S peak |
| Maximum peak distance (range)[ms] | 25-50 | 25-100 | 150-200 |  | 150-320 | 50-80 | 35-65 | 50-125 | 100 | 15-50 |

Abbreviations: P1 and P2 stand for the beginning and the end of the P wave duration. T1 and T2 represent starting and ending points of the T wave. Q, S, P, and T correspond to the local extremes of characteristic points in ECG waveform. NOTE: One value within range was selected for each subject.

In respect to the detected R peak, other peaks were found as the local minimum or maximum on a pre-defined time interval (see Table 1) and stored for further processing with corresponding absolute amplitude (relative to 0 V) (Cabra et al., 2018). For maximum peak distances, we used values within ranges (selected value varied for different groups of subjects) reported in Table 1. All obtained values for characteristic peaks in all subjects (P, Q, R, S, and T) at with-in-beat locations were consistent with the ranges in literature (Palhares et al., 2017; Rawshani, n.d.). The first author performed a visual inspection of detected fiducial points from Table 1 as suggested (Koelsch et al., 2012) and with criteria similar to previously published (Cabra et al., 2018). In the course of visual examination, it was decided to discard ECG from one subject due to the presence of ventricular bigeminy, so further analysis was performed on 70 subjects instead of 71.

Computed ECG peak locations and corresponding absolute peak amplitudes were employed for extracting three groups of clinically relevant and clinically non-relevant features based on the Heart Rate Variability (HRV), temporal parameters, and relative amplitude. An overview of all extracted features is presented in Tables 2-4.

## HRV-based features

HRV is regulated by the autonomic nervous system: HRV increases during relaxation as a result of parasympathetic activity and decreases during stress in relation to sympathetic activity. In the frequency domain, low parasympathetic activity is characterized by the decrease in the high-frequency band and by the increase in the low-frequency band of HRV (Abbasi, 2004). In HRV analysis, R peak locations are used to compute a set of different features. We used three domains to calculate HRV-based features: time, frequency, and geometry. The overview of all extracted features is displayed in Table 2 together with the relevant references related to the application and calculation of HRV-based parameters.

For the computation of HRV-based parameters in the frequency domain, a Power Spectral Density (PSD) of the RR series was estimated. Before the PSD estimation RR intervals were interpolated by the cubic spline interpolation as HRV presents irregularly sampled data (Jang et al., 2012). To calculate PSD, we used a simple nonparametric method – squared Fast Fourier Transformation (FFT).

All HRV-based features were classified as clinically relevant features, except for the HRV index, as it has been defined and consequently used for 24 h ambulatory ECG monitoring and not for short-term recordings of 2 min duration as applied here (Cripps et al., 1991; Kouidi et al., 2002).

TABLE 2 Heart Rate Variability (HRV) based features for three feature domains (time, frequency, and geometry) with corresponding units and related references.

| Feature | Feature Domain | Unit | References | Description |
| --- | --- | --- | --- | --- |
| HR mean | Time | bpm | (Abadi et al., 2015; Kim & Andre, 2008; Tulppo et al., 1996) | Average heart rate |
| RR mean | Time | s | (Abadi et al., 2015; Dissanayake et al., 2019; Electrophysiology, 1996; Kim & Andre, 2008) | Average of all RR intervals |
| rmssd | Time | s | (Abadi et al., 2015; Abbasi, 2004; Dissanayake et al., 2019; Electrophysiology, 1996; Kim & Andre, 2008) | Root mean square of all RR intervals |
| sdnn | Time | s |  | Standard deviation of all RR intervals. |
| m_nn | Time | s | (Abadi et al., 2015; Dissanayake et al., 2019; Kim & Andre, 2008; Koelsch et al., 2012) | Maximal RR interval |
| nn50 | Time | count | (Abadi et al., 2015; Abbasi, 2004; Dissanayake et al., 2019; Electrophysiology, 1996; Kim & Andre, 2008) | Number of pairs of adjacent RR intervals differing by more than 50 ms in the entire recording |
| pnn50 | Time | % |  | nn50 count divided by the total number of all RR intervals |
| sdsd | Time | s | (Abadi et al., 2015; Abbasi, 2004; Electrophysiology, 1996; Kim & Andre, 2008; Tulppo et al., 1996) | Standard deviation of differences between adjacent RR intervals |
| HRV index | Time | n.u. | (Abbasi, 2004; Cripps et al., 1991; Electrophysiology, 1996; Kouidi et al., 2002) | HRV triangular index - integral of the density distribution (the number of all RR intervals) divided by the maximum of the density distribution at a discrete scale of 1/*fs* bins, where *fs* is a sampling frequency |
| LF | Frequency | s^2^ | (Abadi et al., 2015; Abbasi, 2004; Dissanayake et al., 2019; Electrophysiology, 1996; Kim & Andre, 2008; Koelsch et al., 2012; Tulppo et al., 1996) | Spectral power of low frequency (0.04 – 0.15 Hz) |
| HF | Frequency | s^2^ |  | Spectral power of high frequency (0.15 – 0.40 Hz) |
| LFHF | Frequency | n.u. | (Abbasi, 2004; Dissanayake et al., 2019; Electrophysiology, 1996; Kim & Andre, 2008; Koelsch et al., 2012; Tulppo et al., 1996) | LF to HF ratio |
| LFnu | Frequency | n.u. | (Abbasi, 2004; Dissanayake et al., 2019; Electrophysiology, 1996) | LF in normalized units in relation to the total power without very low frequencies |
| HFnu | Frequency | n.u. |  | HF in normalized units |
| Total Power | Frequency | s^2^ |  | Total PSD power |
| SD1 | Geometry | s | (Dissanayake et al., 2019; Kim & Andre, 2008; Koelsch et al., 2012) | Length of the transverse line of the Poincaré plot in the perpendicular direction. Poincaré plot presents a scatter plot of the current RR interval in relation to the prior RR interval. |
| SD2 | Geometry | s |  | Length of the longitudinal line of the Poincaré plot in the perpendicular direction. |

Abbreviations: n.u. stands for no unit and bpm for beats per minute.

## Temporal ECG features

Temporal features were split into two groups – clinically relevant and non-clinical parameters. Although non-clinical parameters do not have proven theoretical background nor diagnostics relevance, their main advantage over standard clinical parameters is robustness to noise as their calculation is based on the detection of local extrema, i.e., fiducial points (Arteaga-Falconi et al., 2016) and proven efficacy in previous studies related to the individual identification (Arteaga-Falconi et al., 2016; Cabra et al., 2018; Israel et al., 2005; Sansone et al., 2013; T.-W. D. Shen et al., 2010). From the previously determined locations of PQRST extremes (Table 1), we determined three time distances. For each distance, 5 statistical features were computed: maximal value, minimal value, mean, median, and standard deviation.

We calculated 8 clinically relevant temporal parameters. QT interval was compensated for the heart rate by calculating the corrected QT interval (*QTc*) according to Bazzet’s formula (Minoretti et al., 2006):

| $QTc= \frac{QT}{\sqrt{RR}}$ | Equation (2) |
| --- | --- |

For all clinically relevant temporal features mean values and standard deviations were calculated (Wagner, 2001). The overview of extracted features is displayed in Table 3.

TABLE 3 Temporal features – clinically relevant and non-clinicalparameters with normal values and ranges where applicable.

| Distance | Description | Features | References | Normal Range [s] |
| --- | --- | --- | --- | --- |
| PR | Measured from the fiducial point P to the R peak | PR_min, PR_max, PR_mean, PR_median, PR_sd | (Cabra et al., 2018; Dissanayake et al., 2019) | na |
| ST | Measured from the fiducial point S to the fiducial point T | ST_min, ST_max, ST_mean, ST_median, ST_sd |  | na |
| QRS | Measured from the fiducial point Q to the fiducial point S | QRS_min, QRS_max, QRS_mean, QRS_median, QRS_sd |  | na |
| PR interval * | Measured from the beginning of the P wave to the beginning of the QRS complex | PRinterval_mean, PRinterval_sd | (Wagner, 2001) | 0.12 - 0.20 |
| PR segment * | Measured from the end of the P wave to the beginning of the QRS complex | PRsegment_mean, PRsegment_sd |  | 0.05 – 0.12 |
| ST  interval * | Measured from the end of the QRS complex to the end of the T wave | STinterval_mean, STinterval_sd |  | 0.42 |
| ST segment * | Measured from the end of the QRS complex to the beginning of the T wave | STsegment_mean, STsegment_sd |  | 0.005 – 0.150 |
| QRS complex * | Measured from the beginning of the QRS complex to the end of the QRS complex | QRScomplex_mean, QRScomplex_sd |  | 0.08 - 0.12 |
| P wave * | Measured from the beginning of the P wave to the end of the P wave | Pwave_mean, Pwave_sd |  | ≤0.12 |
| T wave * | Measured from the beginning of the T wave to the end of the T wave | Twave_mean, Twave_sd |  | 0.10 - 0.25 |
| QTc interval * | Measured from the beginning of the QRS complex to the end of the T wave and compensated according to Bazzet’s formula | QTnorm_mean, QTnorm_sd |  | Men:< 0.45 Women:< 0.46  0.35 – 0.43 (QT) |

Abbreviations: na – not available, QTc – corrected QT interval. Suffixes _min, _max, _mean, _median and _sd stand for minimal value, maximal value, mean, median, and standard deviation, respectively. NOTE: * stands for clinically relevant parameters

## Amplitude-based ECG features

From the absolute amplitudes of fiducial points (P, Q, R, S and T) relative differences between them were computed (see Table 4). For all relative amplitudes mean values and standard deviations were calculated.

Ek parameter has been suggested as a cardiac signature of emotionality and personality in previous studies (Koelsch et al., 2007, 2012). This ECG-based index presents a weighted linear relation of ECG amplitudes that is unrelated to the person’s BMI, weight, and height and has proven its usefulness and direct correlation with emotionality. Thus, higher Ek indexes correspond to higher emotionality measured by the Revised Toronto Alexithymia Scale (Taylor et al., 1992) and vice versa.

Originally, Ek indices are determined from the 12-lead resting ECG (Koelsch et al., 2007, 2012). By careful studying of the proposed Ek formula and its practical significance (BMI and electrode positioning compensations), we concluded that Ek can be calculated for one-channel ECG. Namely, BMI compensation from the original formula was kept as we did not change the formula itself and the relative ratio of amplitude parameters remained (Eq. 3). In our study compensation of electrode positioning introduced by 12-lead ECG recordings was unnecessary, as we placed electrodes on the same anatomical positions in all subjects. Therefore, only one channel was sufficient. The modified formula for Ek index adopted for one channel ECG recording is:

| $Ek= \alpha\times\frac{Tampl \times Rampl}{{RSampl}^{2}}$ | Equation (3) |
| --- | --- |

where *Tampl* is the amplitude of the T wave, *Rampl* amplitude of the R wave, and *RSampl* presents the relative difference between R and S amplitudes. For better readability of Ek values, values are scaled by a factor *α* = 10 as proposed elsewhere (Koelsch et al., 2007, 2012). The beginning of the P wave was considered as an isoelectric line for measurement of R wave amplitude (Koelsch et al., 2012) and T amplitude was measured as the relative difference from the beginning of the T wave to the T wave peak (Koelsch et al., 2007). Furthermore, mean values and standard deviations of Ek were calculated. It should be noted that Ek parameters were calculated from the ensemble-averaged ECG signal.

TABLE 4 Amplitude-based ECG parameters

| Distance | Feature [n.u.] | References | Description |
| --- | --- | --- | --- |
| PRa | PRa_mean, PRa_sd | (Cabra et al., 2018), MNUA | Relative amplitude differences between P and R |
| RQa | RQa_mean, RQa_sd | (Arteaga-Falconi et al., 2016; Cabra et al., 2018) | Relative amplitude differences between R and Q |
| RSa | RSa_mean, RSa_sd | (Arteaga-Falconi et al., 2016; Cabra et al., 2018) | Relative amplitude differences between R and S |
| RTa | RTa_mean, RTa_sd | (Cabra et al., 2018), MNUA | Relative amplitude differences between R and T |
| STa | STa_mean, STa_sd | (Cabra et al., 2018), MNUA | Relative amplitude differences between S and T |
| QSa | QSa_mean, QSa_sd | (Cabra et al., 2018), MNUA | Relative amplitude differences between Q and S |
| Ek | Ek_mean, EK_sd | (Koelsch et al., 2007, 2012) | Calculating formula is available in Eq. (3) |

Abbreviations: n.u. stands for no unit and MNUA for Mentioned in literature Not Used for Analysis. Ek parameter presents amplitude measure of cardiac personality and emotionality.

# References

Abadi, M. K., Correa, J. A. M., Wache, J., Heng Yang, Patras, I., &Sebe, N. (2015). Inference of personality traits and affect schedule by analysis of spontaneous reactions to affective videos. *2015 11th IEEE International Conference and Workshops on Automatic Face and Gesture Recognition (FG)*, 1–8. https://doi.org/10.1109/FG.2015.7163100

Abbasi, W. A. (2004). *Time Series Analysis of Heart Rate Variability(HRV)Signals*. https://doi.org/10.13140/RG.2.2.30988.33921

Antić, M., Popović, N., Milosavljević, N., Dubljević, O., Bjegojević, B., &Miljković, N. (2020). CardioPRINT: Individual features hidden in electrocardiogram and impedance-cardiogram. *Proceedings of the XXVI Scientific Conference on Empirical Studies in Psychology*, 13–16.

Arteaga-Falconi, J. S., Al Osman, H., & El Saddik, A. (2016). ECG Authentication for Mobile Devices. *IEEE Transactions on Instrumentation and Measurement*, *65*(3), 591–600. https://doi.org/10.1109/TIM.2015.2503863

Augie, B., Antonov, A., &Auguie, M. B. (2017). *gridExtra: Miscellaneous functions for “grid” graphics*. R package version, 2(1). https://CRAN.R-project.org/package=gridExtra

Borchers, H. W. (2019). *Package ‘pracma’: Practical numerical math functions*. R package version, 2(1). https://CRAN.R-project.org/package=pracma

Cabra, J.-L., Mendez, D., & Trujillo, L. C. (2018). Wide Machine Learning Algorithms Evaluation Applied to ECG Authentication and Gender Recognition. *Proceedings of the 2018 2nd International Conference on Biometric Engineering and Applications - ICBEA ’18*, 58–64. https://doi.org/10.1145/3230820.3230830

Cripps, T. R., Malik, M., Farrell, T. G., &Camm, A. J. (1991). Prognostic value of reduced heart rate variability after myocardial infarction: Clinical evaluation of a new analysis method. *Heart*, *65*(1), 14–19. https://doi.org/10.1136/hrt.65.1.14

Developers. (2013). *Signal: Signal processing*. http://r-forge.r-project.org/projects/signal

Dissanayake, T., Rajapaksha, Y., Ragel, R., &Nawinne, I. (2019). An Ensemble Learning Approach for Electrocardiogram Sensor Based Human Emotion Recognition. *Sensors*, *19*(20), 4495. https://doi.org/10.3390/s19204495

*ECG Analysis using the Offline Averaging Mode*. (2006). BIOPAC Systems, Inc. https://www.biopac.com/wp-content/uploads/app177.pdf

Electrophysiology, T. F. of the E. S. of C. the N. A. (1996). Heart Rate Variability: Standards of Measurement, Physiological Interpretation, and Clinical Use. *Circulation*, *93*(5), 1043–1065. https://doi.org/10.1161/01.CIR.93.5.1043

Hadley, W. (2016). *Ggplot2: Elegrant graphics for data analysis*. Springer.

Harrell, F. E. (2017). *with contributions from Charles Dupont and many others.“Hmisc: Harrell Miscellaneous.”* R package version 4.0-1. https://CRAN.R-project.org/package=Hmisc

Israel, S. A., Irvine, J. M., Cheng, A., Wiederhold, M. D., &Wiederhold, B. K. (2005). ECG to identify individuals. *Pattern Recognition*, *38*(1), 133–142. https://doi.org/10.1016/j.patcog.2004.05.014

Jang, D.-G., Minsoo Hahn, Jae-Keun Jang, Farooq, U., & Seung-Hun Park. (2012). A comparison of interpolation techniques for RR interval fitting in AR spectrum estimation. *2012 IEEE Biomedical Circuits and Systems Conference (BioCAS)*, 352–355. https://doi.org/10.1109/BioCAS.2012.6418424

Kim, J., & Andre, E. (2008). Emotion recognition based on physiological changes in music listening. *IEEE Transactions on Pattern Analysis and Machine Intelligence*, *30*(12), 2067–2083. https://doi.org/10.1109/TPAMI.2008.26

Kligfield, P., Hancock, E. W., Helfenbein, E. D., Dawson, E. J., Cook, M. A., Lindauer, J. M., Zhou, S. H., &Xue, J. (2006). Relation of QT Interval Measurements to Evolving Automated Algorithms from Different Manufacturers of Electrocardiographs. *The American Journal of Cardiology*, *98*(1), 88–92. https://doi.org/10.1016/j.amjcard.2006.01.060

Koelsch, S., Enge, J., &Jentschke, S. (2012). Cardiac Signatures of Personality. *PLoS ONE*, *7*(2), e31441. https://doi.org/10.1371/journal.pone.0031441

Koelsch, S., Remppis, A., Sammler, D., Jentschke, S., Mietchen, D., Fritz, T., Bonnemeier, H., & Siebel, W. A. (2007). A cardiac signature of emotionality: A cardiac signature of emotionality. *European Journal of Neuroscience*, *26*(11), 3328–3338. https://doi.org/10.1111/j.1460-9568.2007.05889.x

Komsta, L., &Novomestky, F. (2015). *Moments, cumulants, skewness, kurtosis and related tests*. R package version, 14. https://CRAN.R-project.org/package=moments

Kouidi, E., Haritonidis, K., Koutlianos, N., &Deligiannis, A. (2002). Effects of athletic training on heart rate variability triangular index: Athletic training and heart rate variability. *Clinical Physiology and Functional Imaging*, *22*(4), 279–284. https://doi.org/10.1046/j.1475-097X.2002.00431.x

Kuhn, M., Wing, J., Weston, S., & Williams, A. (2020). *Package ‘caret.’* The R Journal, 223. https://CRAN.R-project.org/package=caret

Meyer, D., Dimitriadou, E., &Hornik, K. (2019). *e1071: Misc functions of the Department of Statistics, Probability Theory Group (e1071), TU Wien.* R package version 1-7. https://CRAN.R-project.org/package=e1071

Minoretti, P., Politi, P., Martinelli, V., Emanuele, E., Bertona, M., Falcone, C., &Geroldi, D. (2006). QT interval duration in apparently healthy men is associated with depression-related personality trait neuroticism. *Journal of Psychosomatic Research*, *61*(1), 19–23. https://doi.org/10.1016/j.jpsychores.2006.01.001

Müller, K., & Wickham, H. (2019). *tibble: Simple Data Frames*. R package version 2.1. 3. https://CRAN.R-project.org/package=tibble

Palhares, D. M. F., Marcolino, M. S., Santos, T. M. M., da Silva, J. L. P., Gomes, P. R., Ribeiro, L. B., Macfarlane, P. W., & Ribeiro, A. L. P. (2017). Normal limits of the electrocardiogram derived from a large database of Brazilian primary care patients. *BMC Cardiovascular Disorders*, *17*(1), 152. https://doi.org/10.1186/s12872-017-0572-8

Paluszynska, A., Biecek, P., Jiang, Y., & Jiang, M. Y. (2020). *Package ‘randomForestExplainer’. Explaining and visualizing random forests in terms of variable importance.* https://CRAN.R-project.org/package=randomForestExplainer

Peterson, B. G., Carl, P., Boudt, K., Bennett, R., Ulrich, J., Zivot, E., &Wuertz, D. (2018). *Package ‘performanceanalytics.’* R Team Cooperation. https://CRAN.R-project.org/package=PerformanceAnalytics

Rawshani. (n.d.). *Clinical ECG Interpretation: Introduction to ECG Interpretation*. https://ecgwaves.com/topic/ecg-normal-p-wave-qrs-complex-st-segment-t-wave-j-point/

Sansone, M., Fratini, A., Cesarelli, M., Bifulco, P., Pepino, A., Romano, M., Gargiulo, F., & Sansone, C. (2013). Influence of QT correction on temporal and amplitude features for human identification via ECG. *2013 IEEE Workshop on Biometric Measurements and Systems for Security and Medical Applications*, 22–27. https://doi.org/10.1109/BIOMS.2013.6656144

Satija, U., Ramkumar, B., & Manikandan, M. S. (2018). A Review of Signal Processing Techniques for Electrocardiogram Signal Quality Assessment. *IEEE Reviews in Biomedical Engineering*, *11*, 36–52. https://doi.org/10.1109/RBME.2018.2810957

Shen, T. W., & Tompkins, W. J. (2005). Biometric Statistical Study of One-Lead ECG Features and Body Mass Index (BMI). *2005 IEEE Engineering in Medicine and Biology 27th Annual Conference*, 1162–1165. https://doi.org/10.1109/IEMBS.2005.1616629

Shen, T.-W. D., Tompkins, W. J., & Hu, Y. H. (2010). Implementation of a one-lead ECG human identification system on a normal population. *Journal of Engineering and Computer Innovations*, *2(1)*, 12–21.

Taylor, G. J., Bagby, M., & Parker, J. D. A. (1992). The Revised Toronto Alexithymia Scale: Some Reliability, Validity, and Normative Data. *Psychotherapy and Psychosomatics*, *57*(1–2), 34–41. https://doi.org/10.1159/000288571

Team, R. C. (2018). *R: A language and environment for statistical computing*. R Foundation for Statistical Computing. https://www.r-project.org

Tulppo, M. P., Makikallio, T. H., Takala, T. E., Seppanen, T., &Huikuri, H. V. (1996). Quantitative beat-to-beat analysis of heart rate dynamics during exercise. *American Journal of Physiology-Heart and Circulatory Physiology*, *271*(1), H244–H252. https://doi.org/10.1152/ajpheart.1996.271.1.H244

Wagner, G. S. (2001). *Marriott’s practical electrocardiography*. Lippincott Williams & Wilkins.

Wei, T., Simko, V., Levy, M., Xie, Y., Jin, Y., &Zemla, J. (2017). *Package ‘corrplot’. Statistician, 56(316), e24*. https://github.com/taiyun/corrplot

Wickham, H., Francois, R., Henry, L., & Müller, K. (2015). *Dplyr: A Grammar of Data Manipulation. R Found. Stat. Comput., Vienna*. R package version 0.4. 3. https://CRAN. R-project. org/package= dplyr.https://CRAN.R-project.org/package=dplyr

Wickham, H., & Pedersen, T. L. (2019). *gtable: Arrange “Grobs” in Tables*. R package version 0.3.0. https://CRAN.R-project.org/package=gtable

Zeileis, A., &Grothendieck, G. (2005). zoo: S3 Infrastructure for Regular and Irregular Time Series. *ArXiv:Math/0505527*. http://arxiv.org/abs/math/0505527
